# Visualisation of magnetic field-induced nanoparticle clusters and mechanical property changes in a breast phantom for inductive moderate hyperthermia

**DOI:** 10.1186/s12938-026-01576-9

**Published:** 2026-04-27

**Authors:** Valerii B. Orel, Alexandr I. Tovstolytkin, Valerii E. Orel, Serhii O. Mamilov, Oleksandra S. Ostapenko, Olga Yo. Dasyukevich, Oleksandr Yu. Rykhalskyi, Sergii A. Lyalkin, Vadim I. Dunaievskyi, Svitlana S. Nazarchuk, Vitaliy Yo. Kotovskyi, Lyudmyla V. Garmanchuk, Olexander Yu. Galkin

**Affiliations:** 1 National Cancer Institute, 33/43 Zdanovska Str., Kyiv, 03022 Ukraine; 2 National Technical University of Ukraine “Igor Sikorsky Kyiv Polytechnic Institute”, 37 Prospect Beresteiskyi, Kyiv, 03056 Ukraine; 3 V.G. Baryakhtar Institute of Magnetism of the National Academy of Sciences of Ukraine, 36-B Akad. Vernadskogo Blvd., Kyiv, 03142 Ukraine; 4 V.E. Lashkaryov Institute of Semiconductor Physics, 45 Nauky Ave., Kyiv, 03028 Ukraine; 5 Taras Shevchenko National University of Kyiv, 4-G Hlushkova Ave., Kyiv, 03022 Ukraine

**Keywords:** Magneto-mechanical effect, Magnetic nanoparticles, Inhomogeneous stationary magnetic field, Inductive moderate hyperthermia, Breast phantom, Medical imaging

## Abstract

**Background:**

Visualising magnetic nanoparticle (MNP) clusters is important for inductive moderate hyperthermia (IMH) as their formation influences mechanical force transduction and heat generation in malignant tumours. An applied inhomogeneous stationary magnetic field (ISMF) can direct the arrangement of MNP clusters and the force exerted on cancer cells. Herein, we analysed MNP cluster formation and changes in mechanical properties using digital breast tomosynthesis (DBT) and ultrasound shear wave elastography (SWE) for a breast phantom containing MCF-7 breast cancer cells under the influence of IMH with ISMF.

**Results:**

Texture analysis of the tumour-mimicking region revealed that ISMF induced a 13% increase in fractal dimension and a twofold decrease in lacunarity of MNP clusters on DBT images, as well as a 20% decrease in lacunarity of apparent stiffness within fixed regions of interest on SWE images, as compared with non-targeted MNPs (p < 0.05). While the addition of MNPs increased the maximum temperature in the tumour-mimicking region only by 1.3 °C, it led to a 3.8-fold decrease in lacunarity and a 10% increase in fractal dimension on thermal images, as well as an 87% lower fraction of viable MCF-7 cells than IMH with ISMF (p < 0.05). We also considered the clinical relevance for breast cancer patients, given that the heat-pain threshold (~ 42 °C) is close to the temperatures observed during IMH, whereas the forces generated by ISMF remain below typical pressure-pain thresholds.

**Conclusions:**

ISMF initiated a more uniform spatial distribution of MNP clusters, altering the SWE-derived apparent stiffness and temperature patterns in the tumour-mimicking region. While causing only a moderate temperature increase (< 42 °C), IMH combination with ISMF and MNPs significantly reduced MCF-7 viability, indicating the additional role of magneto-mechanical effects.

**Supplementary Information:**

The online version contains supplementary material available at 10.1186/s12938-026-01576-9.

## Background

Breast cancer is expected to cause 3.2 million new cases and 1.1 million deaths among women worldwide per year by 2050 [1, 2]. Results from previous clinical trials have indicated higher response and survival rates in patients with advanced and recurrent breast cancer after chemotherapy combined with regional inductive moderate hyperthermia (IMH) (≤ 42 °C) than in those receiving chemotherapy alone [3, 4]. IMH is a non-invasive technique in which an external radiofrequency electromagnetic field generated by a loop applicator induces moderate heating within the tumour and its microenvironment. IMH is used for relatively small target volumes, such as a primary tumour, a group of regional lymph nodes, or a solitary metastasis [5]. In most cases, hyperthermia-based treatment relies on increasing power until the temperature inside the tumour reaches a tolerable maximum to kill cancer cells. Nevertheless, limitations to this approach still remain associated with the inherent heterogeneity of malignant tumours and appreciable temperature rises in the surrounding tissues [6].

Combining IMH with magnetic nanoparticles (MNPs) allows for localised heating as MNPs can be remotely targeted to the tumour site using external stationary magnetic fields that penetrate the human body with minimal attenuation [7, 8]. It is more practical to direct the spatial arrangement of MNPs by applying an inhomogeneous stationary magnetic field (ISMF), where the magnetic field gradient predominantly determines the force on the nanoparticles and thus governs their guidance and clustering into user-designed patterns [9]. Moreover, MNPs transduce mechanical forces to biological media in response to ISMF, otherwise known as the magneto-mechanical effect. MNP-generated forces on the order of 0.2–100 pN are sufficient to activate ion channels and membrane receptors, as well as alter membrane permeability [10–13]. While many membrane channels are sensitive to the mechanical forces, they can also transform other physical stimuli, such as electric, temperature and light, into cellular responses. Different forms of energy are interconverted in cells. For instance, stretching the membrane bilayer produces a change in the voltage dependence of ion channels [14, 15].

Mechanochemical transduction, a process through which cells convert mechanical stimuli into biochemical signals, plays a critical role in breast cancer cell growth and death [16, 17]. MNPs have been found to switch reactive oxygen species (ROS)-dependent pathways by applying mechanical stress on the tumour and its microenvironment under the influence of ISMF. Absorption of electromagnetic field (EMF) energy in the kilohertz (kHz) range is often exploited in IMH for immediate thermal and mechanical destruction of cell integrity by MNPs, whereas the applied field in the megahertz (MHz) range enables modulating intracellular ROS levels [18, 19].

Image-guided treatment planning is a subject of current debate and perspective on the personalised application of IMH to clinical oncology. Patient-specific plans require determining MNP aggregation patterns and temperature distributions within the tumour and the surrounding tissues [20]. However, no such clinical model exists enabling robust, validated and commercially available IMH planning to date. Since obtaining whole samples of human tissue to work with is difficult for clinical and ethical reasons, breast phantoms seeded with cancer cells are needed to realise the incorporation of magneto-mechanical effects into treatment planning.

Previous studies have focused most of their attention on temperature fluctuations within phantoms during IMH alone or in combination with a stationary magnetic field [20–24] rather than the relationship between MNP cluster formation, magneto-mechanical and thermal effects. The above considerations motivate the current work, in which we visualised and analysed MNP cluster formation as well as mechanical property changes for a breast phantom containing MCF-7 cells under the influence of IMH combined with ISMF using medical imaging techniques: digital breast tomosynthesis (DBT) and ultrasound shear wave elastography (SWE). This additional quantitative information is expected to help identify parameters in treatment planning for breast cancer patients with regard to the magneto-mechanical effects of MNPs during IMH.

## Results

### Analysis of MNP clustering

**Fig. 1:**
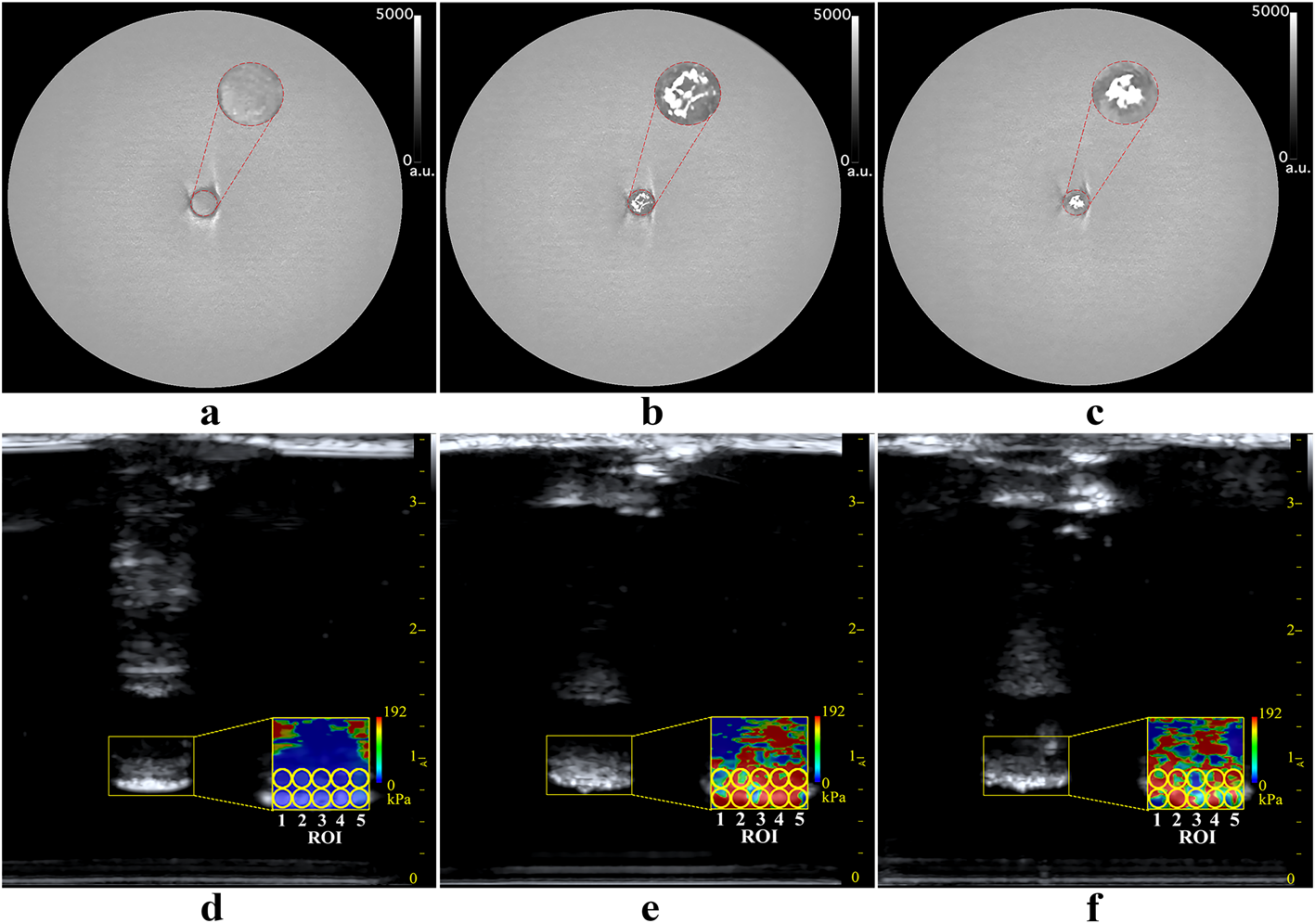
Representative DBT (**a**, **b**, **c**; red circle insets show magnified view of the tumour-mimicking region) and US (**d**, **e**, **f**; yellow box insets show magnified view of SWE-derived apparent stiffness distribution in five circular ROIs placed at 1 mm and 2 mm depth across the bottom of the tumour-mimicking region) images of designed breast phantom: tumour-mimicking region (**a**, **d**); tumour-mimicking region + MNPs (**b**, **e**); tumour-mimicking region + MNPs + ISMF (**c**, **f**)

Figure 1 shows an example of DBT (top row) and US (bottom row) images for the designed breast phantom. MCF-7 cells with DMEM in the tumour-mimicking region produced a slightly more radiolucent area than the bulk breast phantom material with out-of-plane artefacts [25] seen at the edges of the capillary and curvilinear areas due to compression (Fig. 1a). US characteristics of the tumour-mimicking region showed a hyperechoic region primarily related to the acoustic impedance mismatch at the interface between the bottom of the capillary and the gelatine phantom with strong specular reflection and additional diffuse scatter from the cell layer; posterior shadowing behind the capillary bottom; a deeper hyperechoic region produced by reverberation artefacts (repeated bright bands) between the transducer and the distal open lumen, where the DMEM-air interface formed a marked impedance mismatch; a hyperechoic band at the top of the image arising from US reflection at the gelatine-air interface (Fig. 1d). At the same time, an increase in stiffness observed deeper at the capillary edges was primarily due to boundary and reflection artefacts, which are commonplace in SWE [26]. The bulk phantom material appeared as a hypoechoic region surrounding the tumour-mimicking region because homogeneous gelatine had low impedance contrast and thus produced weak backscatter [27]. Adding MNPs to the tumour-mimicking region resulted in diffuse and linear radiopaque densities with an average cluster diameter of 410 ± 30 µm (Fig. 1b), while US examination (Fig. 1e) demonstrated a more granular and hyperechoic texture at the bottom of the tumour-mimicking region with MNPs (154.60 ± 0.48 a.u.) than without them (146.33 ± 0.55 a.u., p < 0.05). MNP clusters were sufficiently large relative to the US wavelength at 5–10 MHz (1.4λ–2.7λ) to enter a non-Rayleigh scattering regime with stronger echoes than those of cells in the medium alone [28]. Submicrometric- to micrometric-sized clusters of iron oxide MNPs are known to form in biological media [29]. In fact, dense MNP clusters, such as nanoparticle swarms, can increase US backscatter, producing a hyperechoic region on B-mode images [30]. High stiffness regions behind MNP clusters corresponded to artefacts associated with a lower signal-to-noise ratio secondary to scattering of US waves from MNPs [31]. In response to ISMF targeting, MNPs appeared as tightly aligned radiopaque densities with an average cluster diameter of 740 ± 70 µm (Fig. 1c), causing more pronounced X-ray beam hardening and scatter (darker streaks between and around MNP clusters) as well as a 2.1-fold increase in pixel density compared with non-targeted MNPs (p < 0.05). The bottom of the tumour-mimicking region displayed the lowest echogenicity (143.89 ± 0.55 a.u., p < 0.05), consistent with greater attenuation of US energy by anisotropic cluster formations [32] when MNPs were aligned by ISMF (Fig. 1f). It has previously been shown that ISMF induces US velocity anisotropy in an aqueous system containing MNPs [33]. Visual comparison of acquired images showed a different spatial pattern of SWE-derived apparent stiffness distribution in regions of interest (ROIs) 1 and 3–5 following ISMF application, while SWE images maintained an artefactual increase in stiffness behind MNP clusters.

Quantitative analysis of tumour-mimicking regions in acquired DBT images (Table 1) revealed that the application of ISMF resulted in a 13% higher median value of fractal dimension, a 2.1-fold increase in the median pixel density and a twice lower median value of lacunarity than in the absence of magnetic targeting (p < 0.05). Given that the tumour-mimicking region without MNPs had no large X-ray contrast inclusions, it exhibited the highest fractal dimension and pixel density, as well as the lowest lacunarity. These changes indicate that ISMF induced a more compact and uniform spatial distribution of MNP clusters.

**Table 1:** Texture parameters of the tumour-mimicking region on DBT images, Median (Q1–Q3)

Experiment	Fractal dimension, a.u	Lacunarity, a.u	Pixel density, pixel^−1^
Tumour-mimicking region	1.78 (1.77–1.78)	0.22 (0.21–0.23)	0.99 (0.98–1.00)
Tumour-mimicking region + MNPs	1.45* (1.44–1.46)	0.47* (0.44–0.49)	0.27* (0.13–0.39)
Tumour-mimicking region + MNPs + ISMF	1.67*^#^ (1.65–1.68)	0.24*^#^ (0.22–0.25)	0.56*^#^ (0.35–0.76)

^*^ significant difference from tumour-mimicking region, p < 0.05;

^#^ significant difference from tumour-mimicking region + MNPs, p < 0.05

Data are shown from three independent biological replicates (n = 3), each consisting of three technical repeats

**Fig. 2:**
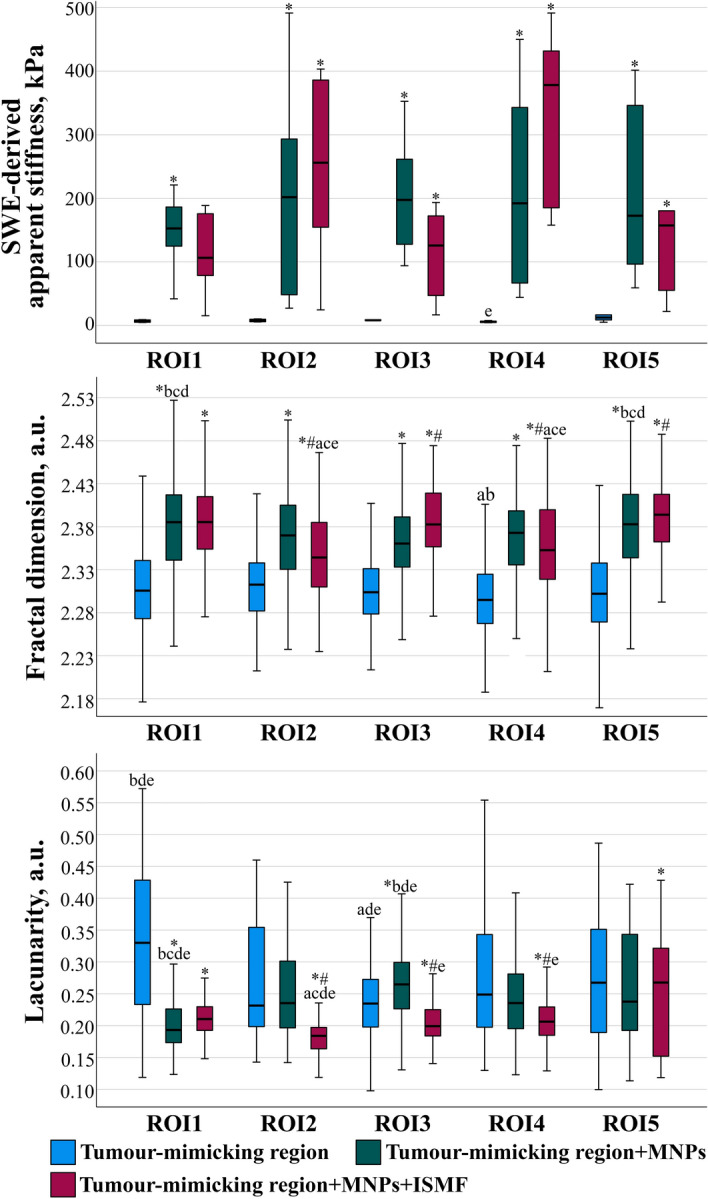
Comparison of apparent stiffness, fractal dimension and lacunarity of the tumour-mimicking region on SWE images. The lower and upper borders of the boxes represent the lower and upper quartiles (25th and 75th percentiles). The middle horizontal lines represent the median. The lower and upper whiskers represent the minimum and maximum values. Data are shown from three independent biological replicates (n = 3), each consisting of three technical repeats. ^*^ significant difference from tumour-mimicking region, p < 0.05; ^#^ significant difference from tumour-mimicking region + MNPs, p < 0.05; ^a^ significant difference from ROI1 of the same experimental condition, p < 0.05; ^b^ significant difference from ROI2 of the same experimental condition, p < 0.05; ^c^ significant difference from ROI3 of the same experimental condition, p < 0.05; ^d^ significant difference from ROI4 of the same experimental condition, p < 0.05; ^e^ significant difference from ROI5 of the same experimental condition, p < 0.05

Quantitative analysis of SWE images was restricted to ROIs at 1–2 mm depth from the bottom of the capillary, as MNPs were not located beyond this depth on CT and no statistically significant differences in the apparent stiffness were observed between tumour-mimicking regions at 3–4 mm depth. As shown in Fig. 2, adding MNPs to the capillary with MCF-7 cells in DMEM led to a 23-fold increase in the median apparent stiffness value, on average (p < 0.05). While ISMF did not produce a significant increase in the median values of apparent stiffness in the tumour-mimicking region with MNPs, fractal and lacunarity analyses revealed distinct changes in its spatial distribution. Prior studies have demonstrated that extracting texture parameters, including fractal dimension and lacunarity, from US elastography images allowed for quantitative characterisation of structural and mechanical heterogeneity in tissues [34, 35]. In this work, the fractal dimension varied across ROIs 2–5 under the influence of ISMF, showing higher median values in ROIs 3 and 5 and lower values in ROIs 2 and 4, as compared with non-targeted MNPs. ISMF caused, on average, a 20% and 17% decrease in median lacunarity within ROIs 2–4, when compared with the corresponding ROIs with and without MNPs, respectively (p < 0.05).

Together, the obtained DBT and US results indicate that ISMF altered the spatial distribution of MNPs and the resulting SWE-derived apparent stiffness pattern in the tumour-mimicking region.

### Analysis of temperature distribution

**Fig. 3:**
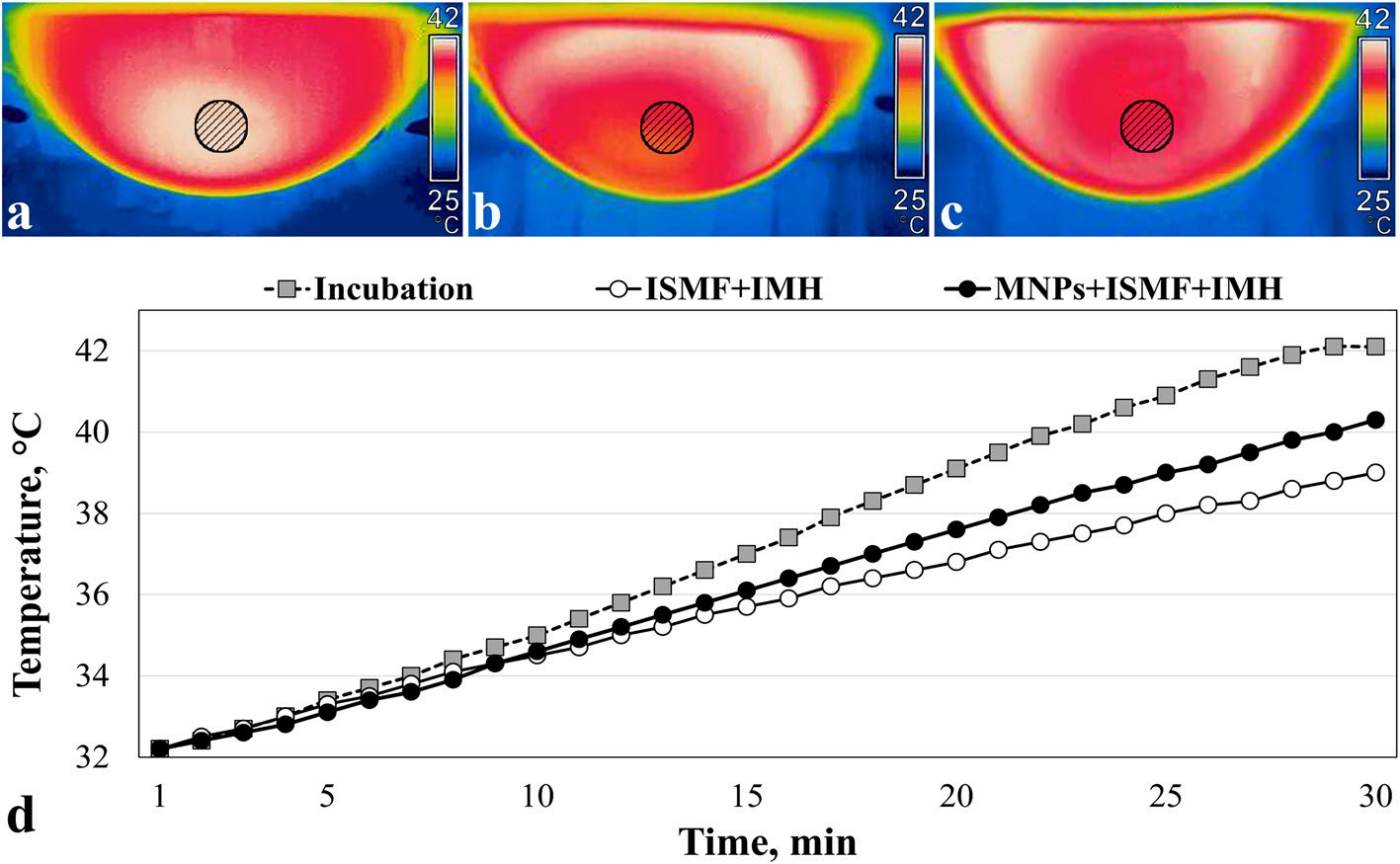
Representative surface temperature distribution images and temperature rise curves shown for tumour-mimicking region within the breast phantom after 30 min exposure to (**a**) incubation (T = 31.809e^0.01t^, R^2^ = 0.9955, T_max_ = 42.1 ºC), (**b)** ISMF + IMH (T = 32.245e^0.0065t^, R^2^ = 0.9963, T_max_ = 39.0 ºC) and (**c**) MNPs + ISMF + IMH (T = 31.908e^0.008t^, R^2^ = 0.9975, T_max_ = 40.3 ºC). Circular regions of interest indicate the projection of the tumour-mimicking region

Figure 3 shows temperature distribution maps on the surface of the breast phantom and temperature rise curves recorded by fibre-optic probes inserted into the tumour-mimicking region after incubation, ISMF + IMH and MNPs + ISMF + IMH. The experimentally measured temperature distribution was consistent with the COMSOL numerical simulation under the incubation condition (Supplementary Figure S2), confirming that the phantom geometry and boundary conditions used in the model adequately reproduced the observed thermal behaviour. Along with previous reports [36, 37], IMH induced a heating pattern with higher temperatures at the edges of the phantom than in its centre, as compared with incubation (Fig. 3a), due to proximity to the loop applicator (Fig. 3b). The presence of MNPs increased the temperature in the tumour-mimicking region by 1.3 ºC as compared with ISMF + IMH (Fig. 2b, c). However, the temperature did not exceed 42 °C either in the tumour-mimicking region or the whole phantom. Although the loop applicator was located beneath the phantom, thermal imaging maps show the surface maximum at the top because of asymmetric boundary conditions (bottom contact with the holder and free top surface), with buoyancy‑driven air convection influencing the apparent pattern. Most hyperthermia studies employ such temperatures since the pain threshold produced by heating in the human chest is ≤ 42 °C on average [38, 39]. In addition, the viability of MCF-7 cells exposed to MNPs + ISMF + IMH was significantly lower than that of ISMF + IMH or incubation (Table 2 and Supplementary Figure S1, p < 0.05).

**Table 2:** Texture parameters of temperature distribution in the tumour-mimicking region and MCF-7 cell viability, Median (Q1–Q3)

Experiment	Fractal dimension, a.u	Lacunarity, a.u	Cell viability, %
Incubation	1.09 (1.07–1.11)	0.24 (0.22–0.25)	96.00 (92.60–97.50)
ISMF + IMH	1.08 (1.05–1.10)	0.15* (0.13–0.17)	92.70 (86.40–96.85)
MNPs + ISMF + IMH	1.19*^#^ (1.19–1.19)	0.04*^#^ (0.04–0.05)	5.40*^#^ (4.35–6.00)

^*^ significant difference from incubation;

^#^ significant difference from ISMF + IMH, p < 0.05

Data are shown from three independent biological replicates (n = 3), each consisting of three technical repeats

Interestingly, MNPs + ISMF + IMH resulted in a more uniform and compact spatial distribution of temperature in the tumour-mimicking region, as determined by a 3.8-fold decrease in lacunarity and a 10% increase in fractal dimension, when compared with ISMF + IMH alone (Table 2, p < 0.05). In agreement with ref [40], in which MNP clustering pattern with a higher fractal dimension enhanced thermal convection flows, the obtained results can be explained by changes in thermal convection. In contrast to a uniform stationary magnetic field, the spatial gradient of ISMF facilitates a gradual transition from natural to thermomagnetic convection, bringing cooler fluid from the capillary walls with higher magnetisation closer to the magnet centre [41].

## Discussion

This study visualised and analysed the formation of MNP clusters as well as changes in mechanical properties within a breast phantom for the application of IMH combined with ISMF. In doing so, we employed DBT and SWE, which are medical imaging techniques approved for diagnostic breast imaging in clinical settings. In the following pages, we discuss the magneto-mechanical and thermal effects initiated by MNPs (Table 3) in the tumour-mimicking region with MCF-7 cells in DMEM under the influence of ISMF and IMH.

**Table 3:** Proposed cascade of magneto-mechanical and thermal effects initiated by MNPs in cancer cells under the influence of ISMF and IMH

Effect		Outcome
A	Movement and aggregation	MNPs are subjected to the magnetic, dipole–dipole, hydrodynamic drag, contact and gravitational forces in the tumour region
B	Cluster formation	Spatial distribution of MNP clusters changes the stiffness in the tumour region under the influence of an inhomogeneous stationary magnetic field
C	Magneto-mechanical transduction	MNP clusters exert mechanical forces on cancer cells, deform cell membranes and activate mechanosensitive ion channels (for example, Piezo and transient receptor potential channels)
D	Mechanochemical transduction	Ca^2+^ influx through open mechanosensitive ion channels results in mitochondrial dysfunction, ROS generation, oxidative stress and cell death
E	Exposure to IMH	EMF induces a moderate temperature increase, altering the cell membrane thickness, clustering pattern of lipid rafts and ion diffusion, while modulating the kinetics of free radical reactions by influencing electron spin dynamics within radical pairs

In biological media, the formation of clusters for ferromagnetic MNPs, which characterises the movement and aggregation (Table 3, Effect A), can be described by Eq. 1 [42].

1 $${m}_{i}\frac{d{v}_{pi}}{dt}={F}_{M}+{F}_{dip}+{F}_{drag}+{F}_{c}+{F}_{m},$$

where ${m}_{i}$ is the mass of MNP *i*, *v*_*pi*_ is the i^th^ MNP’s velocity, *F*_*M*_ is the magnetic force, *F*_*dip*_ is the dipole–dipole force between MNPs, *F*_*drag*_ is the hydrodynamic drag force experienced in living cells or blood and lymphatic vessels, *F*_*c*_ is the contact force and *F*_*m*_ is the gravitational force.

While we mainly focused on the magnetic force resulting from ISMF in the range ∼10^–9^–10^–12^ N (Supplementary Table S1), MNPs experience a large number of other interactions, such as dipole–dipole interactions (∼10^–15^ N), hydrodynamic interactions (∼10^–15^–10^–14^ N), contact interactions (∼ 10^–10^–10^–13^ N) and gravitational interactions (∼10^–18^ N), as detailed in refs [13, 43–45]. These estimates indicate that, under our experimental conditions, the magnetic force generated by ISMF was the dominant driving force governing MNP motion when compared with other interactions.

Cluster formation (Table 3, Effect B) in MNP suspensions occurs when MNPs interact with each other. Provided that the attractive forces between MNPs become stronger than the repulsive forces, they remain bound together. The magnetic force tends to orient the magnetic dipole moments of MNPs along the external magnetic field lines. As a result, each MNP develops an induced magnetic field and behaves as a secondary magnetic dipole that can be represented as a pair of north and south magnetic poles. Depending on their relative orientation, neighbouring MNPs may experience either attraction or repulsion due to dipole–dipole interactions (*F*_*dip*_). Even in the absence of an external magnetic field, ferromagnetic MNPs are prone to forming chain and ring clusters due to dipolar magnetic interactions. ISMF contributes to these attractive interactions by exerting the magnetic force that directs MNPs towards regions of the strongest field strength; the magnetic attraction between MNPs in the surrounding medium increases proportionally with the square of field intensity [46]. Prior studies demonstrated that sub‑second ordering of MNPs occurred in fluid suspensions in response to mT-range magnetic fields [47, 48]. The resulting shape of MNP clusters depends on their remanent magnetisation and the direction of the applied field vector. If the ISMF vector is parallel to the magnetisation direction of MNPs, they tend to align with the dipolar field and converge. Conversely, if the configuration is antiparallel, MNP clusters display a pattern in which they orient with the opposite field direction and diverge from one another. In this case, more complex nonlinear structures and branching of MNP clusters are expected to form [9]. As shown in Fig. 1b, c and Table 1, the spatial distribution of MNP clusters became more compact (higher pixel density and fractal dimension) and uniform (lower lacunarity) under the influence of ISMF, which affected SWE-derived apparent stiffness in the tumour-mimicking region (Fig. 2). Cluster formation, in particular, in MNPs with weak magnetic anisotropy and low spatial heterogeneity, can also enhance thermal effects due to more efficient energy absorption and dissipation [49].

According to [50], we consider the intensity *I* and pressure *p* of mechanical waves generated by a single MNP with the radius *R,* volume *V*, magnetisation *M* exposed to ISMF $\overrightarrow{\mu {H}_{DC}}$ and EMF with the frequency *f* and field gradient $\overrightarrow{\nabla }$ *B(t)* in a biological medium with the viscosity *η* given by Eqs. 2–4. This mechanical energy transfer from oscillating or translating MNPs to the cellular microenvironment represents the magneto-mechanical transduction (Table 3, Effect C), during which mechanical stimuli can deform the membrane of nearby cells and activate mechanosensitive ion channels, such as Piezo1.

2 $${v}_{stat}=\frac{VM\nabla {B}_{max}}{6\pi \eta R}$$

3 $$I={v}_{stat}^{2}\cdot {\rho}_{medium}\cdot s$$

4 $$p={v}_{stat}\cdot {\rho}_{medium}\cdot s$$

where *v*_*stat*_ is the stationary velocity of MNP, ${\rho}_{medium}$ is the density of a biological medium and *s* is the speed of mechanical wave propagation in the medium.

There is a clear distinction in mechanical properties and sensitivity between normal cells and cancer cells. Reduced cell viability can be one of the factors to consider when interpreting the differences in apparent stiffness shown in Fig. 2, as the biomechanical properties of MCF-7 cells change if they undergo apoptosis or necrosis [51, 52]. Moreover, MCF-7 breast adenocarcinoma cells display lower apparent stiffness and higher expression of the Piezo1 mechanosensitive channel than MCF10A normal breast epithelial cells [53, 54]. MCF-7 cells also demonstrate greater resistance to bulk heating when compared with other breast cancer cell lines [55]. Unlike transient receptor potential (TRP) channels, Piezo channels switch their conformational state between closed and open in response to mechanical stimuli while remaining largely unaffected by other physical stimuli [56]. Under hyperthermic conditions, however, temperature increases (< 42 °C) can indirectly modulate the gating of Piezo channels by altering the thickness of the lipid bilayer, clustering pattern of lipid rafts in the cell membrane and ion diffusion in the surrounding medium [57].

Another factor contributing to Piezo1 opening is cooperative interactions between ion channels; these interactions depend on the clustering pattern of Piezo1 channels [58]. It was shown that a stiffer cell substrate (25 kPa) was associated with Piezo1 overexpression and Ca^2+^ influx, which subsequently led to ROS generation, increased mitochondrial permeability and cell death via apoptosis, in contrast to cells on a softer substrate (1 kPa) [59–62]. Signalling cascades initiated by mechanically activated Piezo1 channel leading to downstream calcium signalling, oxidative stress and cell death are characteristic of mechanochemical transduction processes (Table 3, Effect D). Nonetheless, we cannot exclude the possibility that spin-regulated electron transport processes between MNPs and the surrounding biological medium contributed to ROS generation in the presence of ISMF and radiofrequency EMF. ISMF causes Zeeman splitting of electron energy levels proportional to the field strength, whereas the applied radiofrequency field provides energy for electron transitions, allowing otherwise forbidden triplet-to-singlet transitions in radical pairs and thereby changing the yields of free radical reactions [63, 64]. In this instance, pH measurements can serve as an indirect indicator of redox processes [65]. MNPs + ISMF + IMH increased pH in the tumour-mimicking region (7.74 ± 0.01) compared with ISMF + IMH (7.55 ± 0.01) and incubation (7.39 ± 0.01), p < 0.05. One strategy to circumvent drug resistance and trigger more breast cancer cell killing is to shift the extracellular pH from acidic to more alkaline [66].

Exposure to IMH (Table 3, Effect E) is accompanied by induction heating, as the applied EMF induces eddy currents in conducting materials such as Fe₃O₄ MNPs [67]. ROS generation is also linked to the thermal response of MNPs under IMH. When ferromagnetic MNPs are assumed to have a spherical shape, it is possible to adopt an equation derived by Smythe to describe the induction heating of a conducting sphere [68]. By applying the number of spheres *n* = *3π/4φR*^*3*^, where *φ* is the volume fraction of MNPs in the medium, we obtain an expression for energy dissipation due to inductive heating per unit volume (Eq. 5) [69, 70]. COMSOL simulations of EMF and SAR distributions in the tumour-mimicking region (Fig. 7, Supplementary Table S2) show that moderate temperature rise induced by IMH can contribute to the proposed cascade (Table 3, Effect E), facilitating thermally mediated cellular responses in combination with magneto-mechanical interactions. In principle, there was good agreement between the internal temperature simulations and the phantom experiments. The maximum temperature inside the tumour-mimicking region differed by less than 5%. The maximum temperature during incubation heating exceeded that achieved with ISMF + IMH or MNPs + ISMF + IMH owing to several factors: the configuration of heating elements inside the equipment, the higher thermal conductivity of DMEM relative to gelatine, the position of the capillary in the phantom [71]. Fractal and lacunarity analyses of thermal maps quantitatively showed that the spatial distribution of MNP clusters affected the spatial heating profile, with higher fractal dimension and lower lacunarity indicating a more compact and uniform temperature pattern (Table 2). An earlier investigation indicated that the Piezo1 channel was involved in sensitising breast cancer cells to hyperthermia treatment by enhancing heat-induced ROS production [72]. Furthermore, exposure to IMH has been reported to induce a moderate temperature increase of up to 40 °C, which in turn affected the kinetics of Fenton and Haber–Weiss reactions in a medium with Fe₃O₄ MNPs, leading to enhanced ROS generation [73].

5 $$P=\frac{9\sigma {\left(\pi {\mu}_{r}{\mu}_{0}fRB\right)}^{2}}{4\varphi \cdot (2{\mu}_{r}^{2}{\mu}_{0}^{2}x+4{\mu}_{r}{\mu}_{0}^{2}{x}^{2}+4{\mu}_{0}^{2}{x}^{3})},$$

where *P* is the total power absorbed by MNP due to induction heating, *σ* is the electrical conductivity of MNP, *R* is the radius of MNP, *μ*_*r*_ is the relative magnetic permeability of MNP in sinusoidal EMF $\overline{H }=\overline{B }/{\mu}_{0}$, *μ*_*0*_ is the magnetic permeability of free space, *x* = *σ /R*, and *φ* is the volume fraction of MNPs in the medium.

Given a substantial body of prior research on the role of ROS-mediated pathways in MCF-7 cell death in response to mechanical and temperature stimuli [74, 75], a comprehensive examination of cell biology was beyond the scope of our work. In terms of limitations, our study results must be interpreted with consideration of the variability in SWE-derived stiffness measurements across commercially available US scanners, due to differences in proprietary algorithms for shear wave generation, tracking and reconstruction. Importantly, previous studies showed that differences in SWE estimates between US machines were much greater in vivo than in vitro [76–78]. To ensure reproducible results, we acquired US scans using the same system and settings. SWE has limitations in heterogeneous media, particularly in the presence of interfaces, inclusions, or mechanical gradients. Magnetomotive ultrasound (MMUS) may serve as a valuable complementary technique because it derives viscoelastic information from magnetically induced displacement of MNP-containing media during a treatment session rather than from shear-wave propagation alone [79]. In addition, although the multimodal design reflects clinically relevant conditions, under which patients with breast cancer are routinely evaluated with different medical imaging techniques [80], each modality is based on different physical principles and characterised by different spatial resolution, contrast and acquisition geometry. We therefore did not expect the absolute values of fractal dimension and lacunarity to fully converge between imaging modalities. Finally, the study was performed with three independent biological replicates, each with three technical repeats, and included only one MNP type in a single in vitro breast cancer model.

Further clinical translation of IMH combined with MNPs should consider safety limits related to heat pain, which depend not only on MNP-mediated heating but also on nonspecific eddy-current heating in exposed tissues. Safety assessment is commonly based on the relationship between the applied field amplitude and frequency; nonetheless, a more conservative recommendation is to avoid local heating above 41 °C, as such temperatures may be associated with discomfort, heat pain and tissue damage [81]. Currently, IMH aims to achieve a moderate temperature range (40–42 °C) in the tumour and the surrounding tissues. However, this target temperature is close to the heat-pain threshold (~ 42 °C) in breast cancer patients [39, 82, 83], whereas the forces generated by ISMF (~ 6.8 × 10⁻^1^⁶ kg/cm^2^ per MNP) are far below typical pressure-pain thresholds (1.1–8.0 kg/cm^2^) [84, 85]. This indicates that the magneto-mechanical effects do not act as a pain stimulus but are mediated through sub-nociceptive cellular and microenvironmental responses, thereby complementing the thermal effects [86].

To verify the proposed cascade of magneto-mechanical and thermal effects, it could be advantageous to conduct direct microscopic visualisation to characterise the behaviour of a small number of MNPs in a phantom under ISMF. Such experiments could help to confirm the dynamics of MNP aggregation and cluster formation predicted in the present study. Furthermore, investigating nanoparticles with different ferromagnetic properties may provide insight into how magnetic anisotropy, saturation magnetisation and MNP size influence the magneto-mechanical cascade. A practical implication is to translate the proposed cascade of magneto-mechanical and thermal effects initiated by MNPs in the phantom with patient-derived cells from tumour biopsy for treatment planning. Future studies should test clinically relevant concentrations of MNPs and evaluate the treatment response of different histologic and molecular subtypes of breast tumours.

## Conclusion

This work used DBT and SWE, which are widely available breast imaging techniques in clinical settings, to visualise and analyse MNP cluster formation and changes in mechanical properties within a breast phantom containing MCF-7 cells for IMH combined with ISMF. Fractal and lacunarity analyses revealed that the application of ISMF resulted in a more uniform spatial distribution of MNP clusters, altering SWE-derived apparent stiffness and temperature patterns in the tumour-mimicking region, as compared with the absence of magnetic targeting. Moreover, the addition of MNPs caused a 1.3 ºC increase in the maximum recorded temperature after 30-min exposure to IMH with ISMF and a lower MCF-7 cell viability than IMH combined with ISMF alone. While most studies primarily focus on achieving moderate heating at 42 ºC, we emphasise the additional role of magneto-mechanical effects initiated by MNPs in further translation of IMH since temperature increases are much closer to the heat pain threshold than the applied forces to that of the pressure pain threshold in breast cancer patients.

## Materials and methods

Each measurement in this study design was used to demonstrate a different physical aspect of the system. DBT imaging visualised the spatial distribution of MNP clusters, SWE quantified the resulting mechanical heterogeneity of the tumour-mimicking region and thermal imaging together with fibre-optic thermometry characterised temperature changes during IMH. COMSOL simulations were used to estimate the distributions of the applied electromagnetic fields and temperature, as well as to support the interpretation of the experimental observations. Importantly, each modality was required to characterise a distinct step of the underlying mechanistic cascade, from nanoparticle redistribution to mechanical response and thermal effects, which cannot be captured by a single technique alone. Altogether, these measurements provide a basis for linking structural, mechanical and thermal changes, supporting the interpretation of the observed treatment effects. Table 4 provides a summary of the devices used to analyse the effects of MNPs.

**Table 4:** Summary of devices used for analysis of MNP effects

Device	Measurement
Hall effect sensor	Magnitude and spatial gradient of an inhomogeneous stationary magnetic field in the phantom region
COMSOL simulations	Magnetic induction, electric field strength, temperature and specific absorption rate during inductive moderate hyperthermia in the phantom
Digital breast tomosynthesis (DBT) mammography system	Size, pixel density, fractal dimension and lacunarity of magnetic nanoparticle clusters on high resolution X-ray images
Computed tomography (CT) scanner	Radiodensity (Hounsfield units) and depth of magnetic nanoparticle clusters within the tumour-mimicking region
B-mode ultrasound (US) and shear-wave elastography (SWE) scanner	Echogenicity (image brightness), SWE-derived apparent stiffness (Young’s modulus), fractal dimension and lacunarity of the tumour-mimicking region on US images
Thermal camera	Surface temperature distribution, fractal dimension and lacunarity of the tumour-mimicking region on thermal images
Fibre-optic thermometer	Local temperature changes in the tumour-mimicking region
Inverted microscope, hemacytometer and spectrophotometer	Dye absorbance, number of viable and non-viable MCF-7 cells

### Breast phantom design and characterisation

We designed a breast phantom to visualise MNP clusters in the medium with tumour cells and their mechanical properties. 24 g of unflavoured gelatine powder (Mriya, Ukraine) was slowly added to a measuring cup with 96 mL of 0.9% NaCl (Nikopharm, Ukraine). The mixture was placed in a 50 °C water bath followed by agitation to release air bubbles. A total volume of 120 mL was poured into a breast-mimicking container (polyethylene hemisphere with a radius of 40 mm, approximating an AA breast cup size). A cylindrical polypropylene capillary (5 mm inner diameter, 7 mm outer diameter, 1 mm wall thickness) was positioned centrally and embedded during casting. The phantom was then cooled overnight at room temperature.

**Fig. 4:**
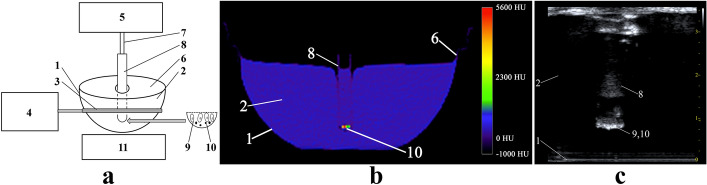
Schematic (**a**), coronal CT (**b**) and coronal view B-mode US (**c**) images of the breast phantom designed to visualise magnetic nanoparticle clusters in the medium with tumour cells and their mechanical properties. Breast-mimicking region: 1—polyethylene container; 2—gelatine; 3—loop applicator; 4—high-frequency generator serving as the source of EMF for IMH; 5—temperature measurement device; 6—cover; 7 – fibre-optic temperature sensor. Tumour-mimicking region: 8 – capillary; 9—MCF-7 cells in DMEM; 10—MNPs; 11 – permanent magnet

Figure 4 shows a schematic, computed tomography (CT) and ultrasound (US) images of the designed breast phantom (n = 3 per experimental condition) consisting of two main compartments: a gelatine matrix representing the breast tissue (breast-mimicking region) and a capillary filled with MCF-7 cells in fluid Dulbecco’s Modified Eagle Medium (DMEM) to mimic a malignant cystic lesion (tumour-mimicking region). Complex cystic breast lesions, i.e., have thick walls, thick septa, or both cystic and solid components, are proven malignant at biopsy in 20–30% of breast cancer patients [87]. MCF-7 cell line was chosen because it is a commonly used in vitro model of luminal A human breast cancer subtype, accounting for 50–60% of all diagnosed breast cancer cases in women [88].

**Fig. 5:**
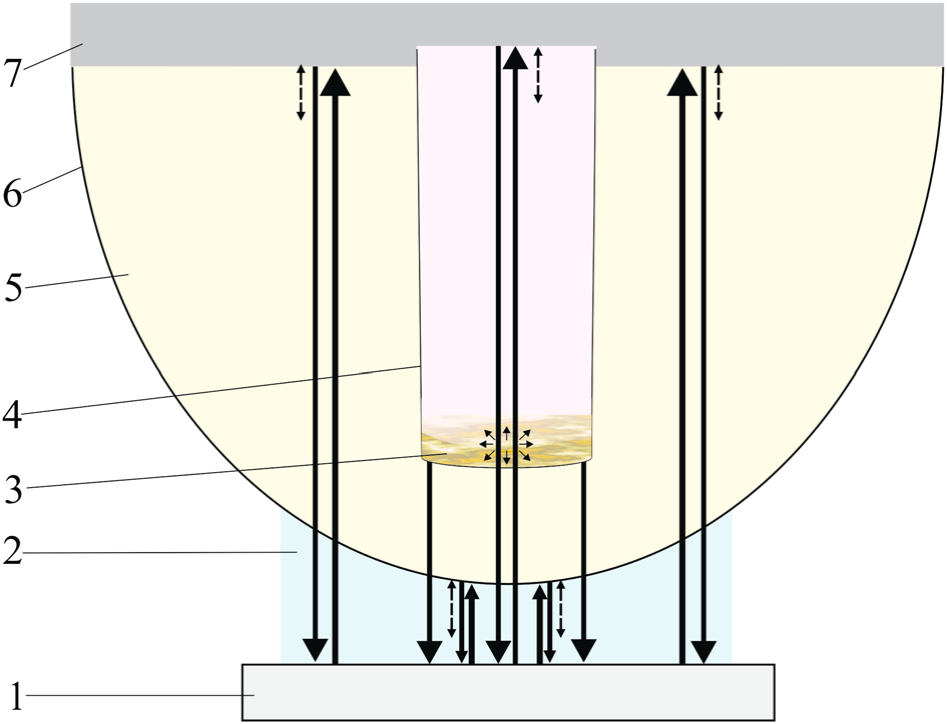
Schematic of US acquisition geometry: 1—linear transducer; 2—coupling gel; 3—MCF-7 cells in DMEM; 4—capillary (tumour-mimicking region) wall; 5—gelatine (breast-mimicking region); 6—phantom wall; 7—air. Solid upward arrows indicate the incident ultrasound beam path (normal incidence; 0° relative to the surface normal of the phantom bottom and the capillary bottom); solid downward arrows indicate reflection paths; dashed bidirectional arrows indicate the repeated segment of the reverberation paths between strong reflecting interfaces (such as the transducer and the bottom phantom wall, or gelatine and air, or capillary filled with cells in DMEM and air); short solid arrows at the bottom of the capillary indicate diffuse scattering from MCF-7 cells. The experimental setup was inverted, with the transducer positioned below the phantom: US beam travelled upwards, encountering the cell layer at the bottom of the capillary as the proximal interface

CT data (350 acquired images) were collected on a SOMATOM go.Top CT system (Siemens Healthineers, Germany) at the Research Department of Diagnostic Radiology (National Cancer Institute of Ukraine) following a standard adult chest protocol (70 kVp, 216 mAs, 0.8 mm slice thickness, 50 cm field of view, 512 × 512 reconstruction matrix). US scanning was carried out with a 6–12 MHz X4—12L linear array transducer (B-mode imaging at 10 MHz, dynamic range of 68 dB, dynamic gain of 30%, mechanical index of 1.6 and thermal index of 0.2, frame rate of 17.2 Hz; SWE acoustic radiation force impulse of 5 MHz, focal depth of the push beam set at 1 cm corresponding to the focus pointer placed below the tumour-mimicking ROI, axial and lateral width of the excitation determined by the 0.5 × 0.5 cm ROI box dimensions, acoustic power of 100%, map dynamic range between 0 and 192 kPa) with post-processing filters (sharpness, speckle reduction, mixing, inertia, grey filter and smoothing) disabled on a Vinno G86 US scanner (Vinno Technology B.V., Netherlands) following SWE recommendations for reliability in a clinical setting based on refs [89, 90]. The transducer was fixed beneath the phantom, perpendicular to the capillary, and secured in a mechanical holder to ensure precise compression. Ultrasound gel was applied to provide effective acoustic coupling and eliminate air gaps between the transducer and the phantom. Although MCF-7 cells were initially in suspension, they formed a dense sediment at the bottom of the capillary driven by intercellular adhesion, which was previously shown to behave as a viscoelastic solid [91, 92]. SWE maps measured the apparent stiffness at the bottom of the capillary, consistent with a cell-rich sediment, yet it was also influenced by wall/boundary conditions and spatial averaging [26]; the DMEM layer above did not support shear waves. The surrounding breast-mimicking region behaved as an elastic soft tissue, whereas the capillary wall introduced a pronounced acoustic–mechanical boundary. Mechanical coupling occurred as shear waves propagated from the surrounding gelatine through the capillary wall and into the MCF-7 layer. Figure 5 shows an idealised schematic with the transducer angle and expected reflection paths during US scanning. Note that the actual US wave behaviour would involve local reflection and refraction in accordance with Snell’s law as the interface has a curved geometry.

Since SWE has methodological limitations and susceptibility to artefacts in heterogeneous media [93], we interpreted the obtained results as SWE-derived apparent stiffness rather than absolute Young’s modulus values. To measure the apparent stiffness of breast-mimicking regions, circular ROIs of 3 mm diameter were placed 3 mm lateral to the lower capillary wall at 1 cm depth, as well as within the phantom region of identical composition that did not contain the capillary. There was no statistically significant difference in SWE-derived apparent stiffness between ROIs in the breast-mimicking region, regardless of the addition of MNPs to the tumour-mimicking region. As shown in Supplementary Table S3, the designed phantom matched to a greater extent the radiodensity and mechanical properties of the breast tissue and malignant breast tumour previously reported in [89, 94, 95]. Adding MNPs led to a significant increase in radiodensity of the tumour-mimicking region on CT (p < 0.05).

### Magnetic targeting of MNPs

**Fig. 6:**
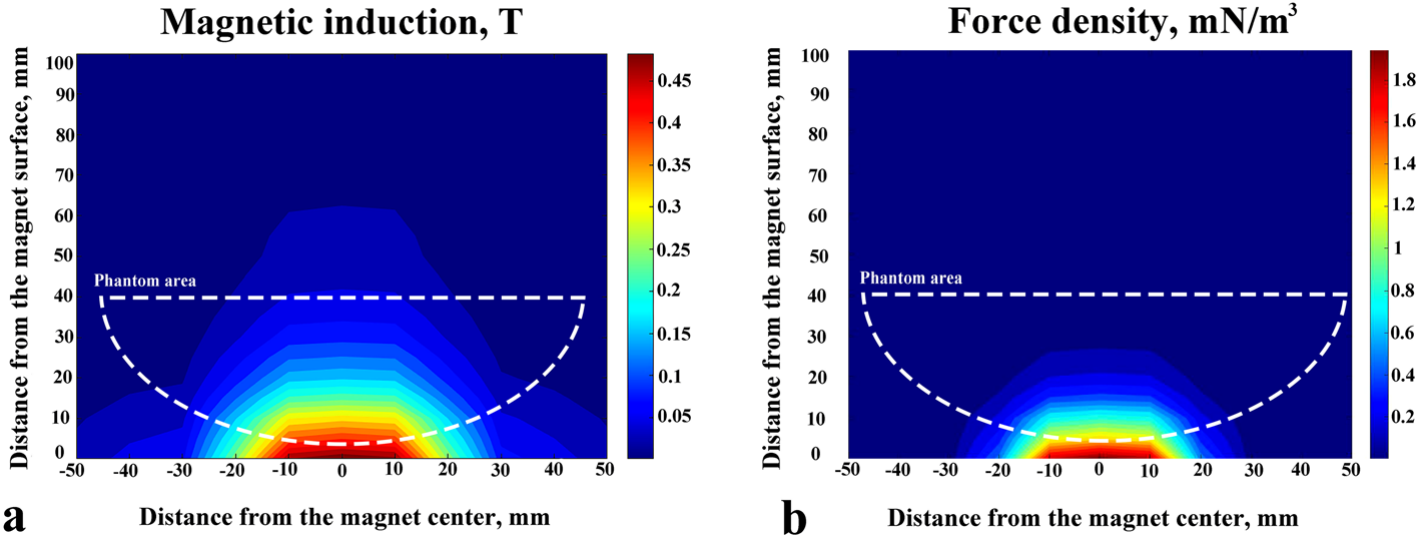
Spatial distribution of magnetic induction (**a**) and magnetic force density (**b**) exerted on a single 50 nm MNP

MNPs used in this study were magnetite (Fe_3_O_4_, Sigma-Aldrich, 637106—25G) with < 50 nm size, magnetic moment of 56.31 emu/g, coercivity of 6.48 Oe and area of the hysteresis loop of 350.95 erg/g. To direct MNP cluster formation and create mechanical forces on MCF-7 cells, a disc Nd_2_Fe_14_B permanent magnet with large coercivity and anisotropy chosen from our previous work [12] was fixed under the tumour-mimicking region such that the centres were matched between the magnet and the base of the phantom (Fig. 4a). The parameters of the magnet are given in Fig. 6 and Supplementary Table S1. We evaluated ISMF inhomogeneity by employing the coefficient of variation method, which measures the dispersion of the magnetic induction in space relative to its mean [96]. As shown in Fig. 6a**,** ISMF had a higher degree of inhomogeneity within < 10 mm from the magnet centre than over a longer distance where the field was weaker. According to [97–100], the spatial distribution of an applied field was measured using a Hall sensor to verify the magnitude and spatial gradient of ISMF in the phantom region, as well as to estimate the magnetic force acting on a single ferromagnetic MNP (Fig. 6b).

### Inductive moderate hyperthermia

An experimental prototype MagTherm device (Radmir, Ukraine) with a loop applicator wired to a high-frequency generator (42 MHz frequency and 10 W output power) served as a source of electromagnetic irradiation for IMH. The applicator’s thermal insulation prevented surface hot spots with minimal radiofrequency interference. A 30-min exposure was chosen because the tumour‑mimicking region reached thermal steady state within this period, consistent with hyperthermia protocols (20–40 min), ensuring safe and clinically relevant conditions [38, 39]. Temperatures measured on the permanent magnet’s surface did not exceed 30 ºC. The distribution of EMF within the breast phantom was modelled in COMSOL Multiphysics (Burlington, MA, USA) v. 5.6 based on [4, 101] to determine optimal parameters for IMH. A key parameter for IMH is the maximum temperature (≤ 42 °C) achieved during a treatment session. There is evidence that side effects are expected to occur at higher temperatures, for example, expression of heat-shock proteins leading to thermoresistance and heat intolerance in patients. With the choice of moderate temperatures, hyperthermia is well tolerated even taking into account the induction field generated near the heart [102, 103]. Our preliminary investigations showed that the shape of MNP clusters modified the specific absorption rate (SAR) distribution in the tumour. In this computational model, MNP clusters were shaped as a combination of right triangles rotated at a 45-degree tilt and merged into a single geometry.

**Fig. 7:**
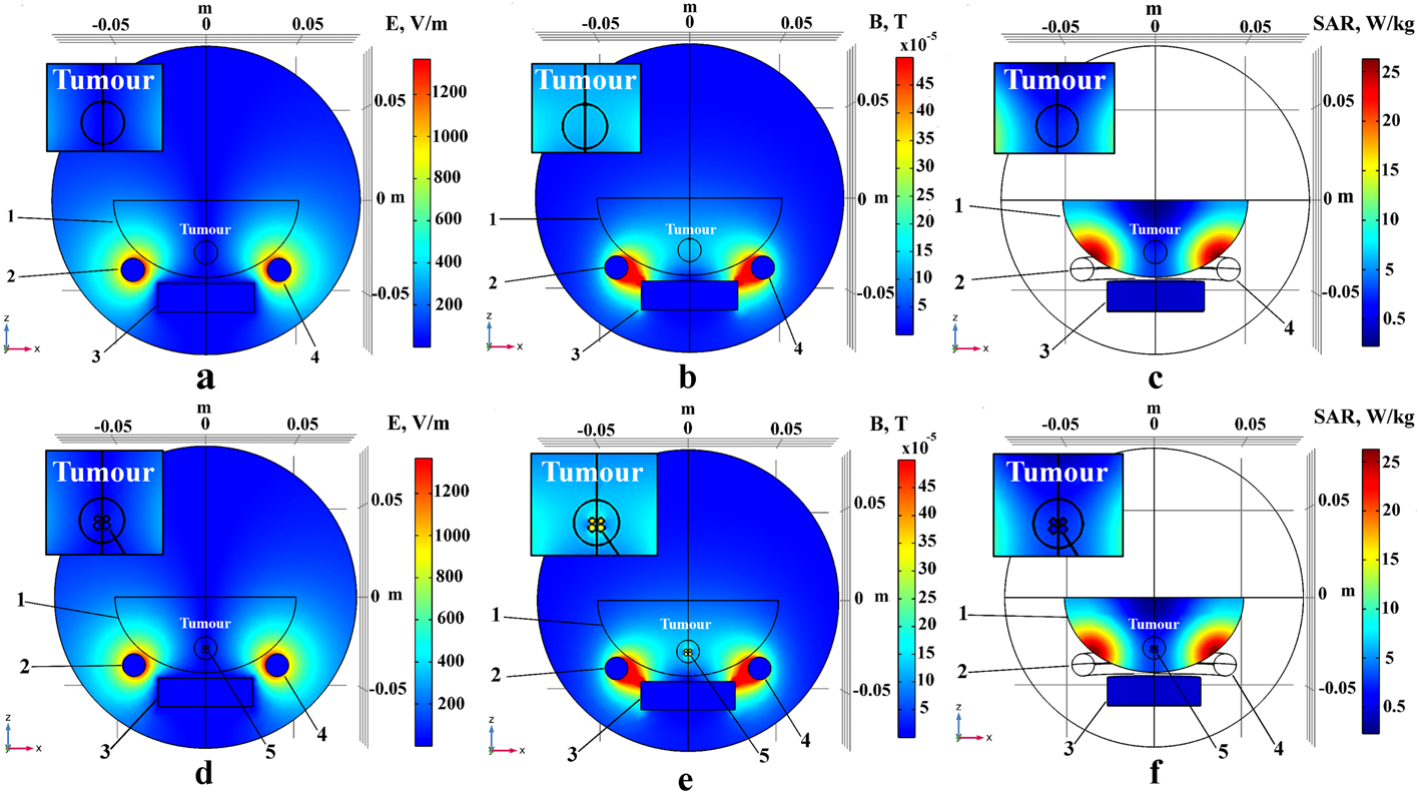
COMSOL simulation of the electric (**a**, **d**), magnetic (**b**, **e**) components and SAR (c, f) in the breast phantom after 30 min IMH session: IMH (**a**–**c**); MNPs + IMH (**d**–**f**). 1—breast phantom; 2, 4—loop applicator; 3—permanent magnet; 5—MNPs. Top-left corner insets show a magnified view of the tumour-mimicking region. the stationary magnetic field produced by the permanent magnet is not included in panels; its distribution is shown separately in Fig. 6

First, we simulated a temperature increase inside the breast phantom after 30 min incubation (Supplementary Figure S2) to establish a baseline thermal distribution and provide a reference for comparison with experimentally measured temperature maps obtained using thermal imaging and fibre-optic temperature measurements. Second, we modelled the distribution of the electric and magnetic components of EMF generated by the MagTherm device and SAR for the phantom in the absence of MNPs (Fig. 7a–c). Magnetic flux density values in the phantom region varied from 3.59 × 10^⁻5^ T to 5.62 × 10^⁻4^ T. Then, COMSOL simulation was run for IMH with MNPs loaded into the tumour-mimicking region (Fig. 7d–f). Supplementary Table S2 summarises the maximum values of the electric field intensity (E, V/m), magnetic induction (B, T), SAR (W/kg) and temperature (T, ºC) calculated from the distributions in Fig. 7.

### MCF-7 cells

MCF-7 human breast cancer cells were cultivated in DMEM (Sigma, USA) with 10% bovine serum (Sigma, USA), 2 mM L-glutamine (Sigma, USA) and 40 μg/mL gentamicin sulphate (Biopharma, Ukraine) under standard conditions (37 °C, 5% CO_2_ and 100% humidity, MM Medcenter Einrichtungen GmbH, Germany). The cells were trypsinised and suspended in a total volume of 0.5 mL DMEM and 9 mg/mL NaCl at 80–90% confluency. The final concentration of the cells was 50 × 10^5^ per 0.5 mL. We then subjected MCF-7 cells within the phantom either to heating incubation at 42 °C using TC-80 M-2 equipment (Ukraine), ISMF + IMH or MNPs + ISMF + IMH for 30 min. MNP concentration of 0.2 mg/mL [104] was added directly to the tumour-mimicking region containing MCF-7 cells without a washing out step, thus MNPs had predominantly extracellular distribution over a 30-min exposure to ISMF + IMH. MCF-7 cell viability was assessed using a standard method with 0.4% trypan blue solution diluted in 0.1 M phosphate-buffered saline at pH 7.2 based on its ability to accumulate only in cells with enhanced membrane permeability and breached membrane integrity using a Labsystem Multiscan MS spectrophotometer (Thermo Fisher Scientific, Korea) and AxioVert inverted microscope (Carl Zeiss) [105]. The number of viable and nonviable cells was counted in a hemacytometer after each treatment. We performed three independent biological replicates (n = 3), each consisting of three technical repeats, which followed commonly accepted standards for in vitro assays [106].

### Visualisation and image analysis

As growing attention has been focused on the need to accelerate the translation of magnetic nanotechnology to cancer theranostics [107], our intention here was to use imaging techniques that are already available and approved for diagnostic imaging, treatment response evaluation and surgical planning of breast cancer patients in the clinical setting. Previously, X-ray and US medical imaging techniques were separately used to visualise MNP clusters and their stiffness in a soft tissue phantom [30, 108, 109].

Quantitative image analysis of MNP cluster formation in the tumour-mimicking region was based on 3D DBT images (120 acquired images). All datasets were acquired using a Giotto Class 30,000 X-ray system (IMS Giotto, Italy) at a tube voltage of 23 kV, 30 mAs, W/Al target filter combination, phantom compression of 40 N, slice thickness of 1 mm and average absorbed dose of 0.24 ± 0.004 mGy.

Changes in mechanical properties of the tumour-mimicking regions were examined via SWE using the X4—12L linear array transducer, Vinno G86 US scanner (Vinno Technology B.V., Netherlands; 130 acquired images), as described above. The ROI template comprised 20 distinct ROIs (each 1 mm in diameter), arranged in four depth levels (1 mm, 2 mm, 3 mm and 4 mm), with five ROIs per depth level spanning across the bottom of the capillary based on CT findings indicating that MNPs were distributed to a depth of ≈2 mm. B-mode US images were visually interpreted before placing box regions of interest (5 × 5 mm) in SWE images.

We used an uncooled thermal camera (ThermaCAM E300, FLIR Systems, USA) with a sensitivity of 0.1 °C within 8–14 μm wavelength bandwidth to monitor temperature distributions (30 acquired images) caused by changes in the tumour-mimicking region of the breast phantom [110, 111]. In addition, MNP effects on the heating pattern were validated by direct fibre-optic measurements (Radmir, Ukraine) in a capillary containing MCF-7 cells and DMEM, placed at the centre of the phantom. The fibre-optic temperature probe was inserted through the polypropylene capillary using a temporary mechanical guiding system consisting of a centring ring with a central aperture and a rigid insertion tube of fixed length, which defined both the insertion axis and depth. The guiding tube was designed as a longitudinally split structure, allowing it to be removed after probe placement without disturbing the fibre position. After probe positioning, all guiding elements were removed prior to SWE and DBT imaging to avoid interference with shear-wave propagation and X-ray acquisition. The capillary remained rigidly embedded within the gelatine phantom and served as a stable positional reference channel. The probe trajectory and location were additionally verified using B-mode ultrasound and DBT, enabling visualisation of linear structures such as capillaries and needle-like probes [112]. Surface infrared thermography was performed with emissivity set to 0.98, under controlled ambient conditions (25 ± 1 °C, 40–60% relative humidity) and calibrated against a 50 °C black body; view-factor errors were minimised (≤ 30° incidence, 0.3–0.5 m distance). Surface maps were co-registered to the fibre-optic probe using fixed fiducials and temperature pattern correlation, with an overall registration error of about ± 1 °C. A fixed 30-min window ensured steady-state, confirmed when temperature fluctuations were <  ± 0.2 °C over the final 5 min. To avoid interference with shear-wave propagation, the fibre-optic probe was removed during DBT and SWE imaging.

Texture parameters, including fractal dimension, lacunarity and pixel density, were extracted from acquired DBT, SWE and thermal images for quantitative characterisation of changes in MNP cluster formation, stiffness and temperature distributions. Circular regions of interest of equal size were drawn around the projection of the capillary in 2D X-ray images or included the lumen of the capillary in 3D X-ray images. ROIs in SWE images were placed within the above-described template. The collected datasets were converted to 8-bit grayscale, segmented by applying the maximum entropy threshold for DBT images, the Canny edge detector for thermal images and the Gray3: Diff Volume Plus 1 method for SWE images, and then analysed using the FracLac plugin in ImageJ 1.53 k (NIH, USA) software. FracLac estimated fractal dimension using the box-counting method by repeatedly covering each ROI with grids of progressively smaller box sizes and analysing how image detail changed with observation scale. The resulting slope of the log–log regression was taken as the fractal dimension (D_B_), with higher values indicating greater structural complexity and compactness of image texture. Lacunarity (Λ) was calculated from the variance of the box mass/intensity distribution across scales, with higher values indicating a more gapped and heterogeneous texture [113–115].

### Statistical analysis

Shapiro–Wilk and Kolmogorov–Smirnov tests determined if the data were normally distributed. Datasets between two groups were compared using the Student's t-test or the Mann–Whitney U-test. Experimental data from three or more groups were compared using one-way ANOVA followed by the post-hoc Tukey or Games-Howell test, or the Kruskal–Wallis test with p < 0.05 considered significant. The coefficient of determination (R^2^) was calculated for exponential regression models of the temperature increase according to the Arrhenius model [116]. All analyses were carried out with SPSS Statistics 25.0 (IBM, Inc., 2017).

## Supplementary Information

Supplementary material 1.

## Data Availability

The datasets used and/or analysed during the current study are available from the corresponding author on reasonable request.

## Abbreviations

CT: Computed tomography
DBT: Digital breast tomosynthesis
DMEM: Dulbecco’s Modified Eagle Medium
EMF: Electromagnetic field
HU: Hounsfield units
IMH: Inductive moderate hyperthermia
ISMF: Inhomogeneous stationary magnetic field
kHz: Kilohertz
MNPs: Magnetic nanoparticles
MHz: Megahertz
SWE: Shear wave elastography
ROS: Reactive oxygen species
ROI: Region of interest
SAR: Specific absorption rate
US: Ultrasound
