# Supplementary material for: Visualisation of magnetic field-induced nanoparticle clusters and mechanical property changes in a breast phantom for inductive moderate hyperthermia
Source: Biomed Eng Online. 2026 Apr 27;25:79. doi: 10.1186/s12938-026-01576-9 (PMC13255342; doi:10.1186/s12938-026-01576-9)
Supplement: Supplementary file 1 — Supplementary material 1. [file 12938_2026_1576_MOESM1_ESM.docx]

**SUPPLEMENTARY INFORMATION**

**
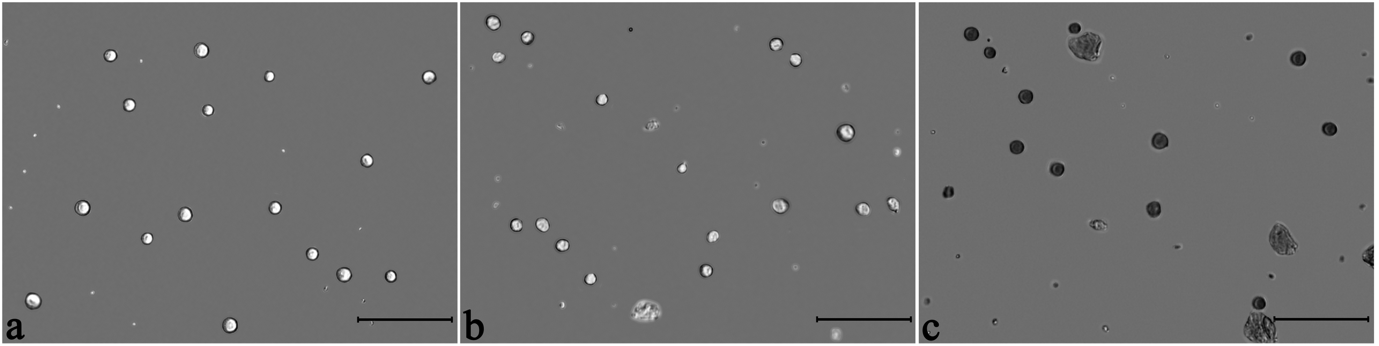
**

**Supplementary Figure S1.** MCF-7 cell viability assay with trypan blue (scale bars representing 25 μm) after 30 min exposure to incubation (a); ISMF + IMH (b), MNPs + ISMF + IMH (c).

**Supplementary** **Table S1.** Parameters of a disc neodymium magnet

| **Diameter, mm** | **Thickness, mm** | **Magnetic induction, T** | | **Inhomogeneity, %** | | **Force on a single MNP, pN** |
| --- | --- | --- | --- | --- | --- | --- |
|  |  | Distance from the magnet centre | | | |  |
|  |  | < 30 mm | > 30 mm | < 30 mm | > 30 mm |  |
| 45 | 15 | 0.017–0.506 | < 0.017 | 78.83 | 70.57 | 0.001–255.28 |


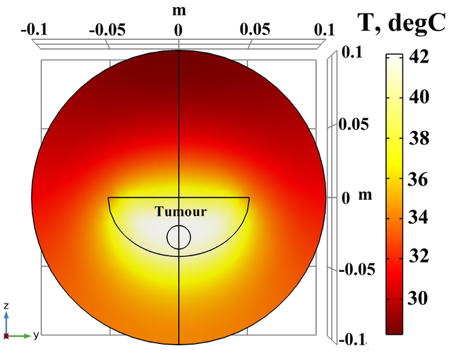


**Supplementary Figure S2.** COMSOL simulation of temperature distribution in the breast phantom after 30 min incubation.

**Supplementary Table S2.** Maximum values of modelled parameters in the tumour-mimicking region

| **E, V/m** | **B, T** | **SAR, W/kg** | **T, ºC** |  |
| --- | --- | --- | --- | --- |
| *IMH* | | | | |
| 32.91 | 1.05·10^-4^ | 8.03 | 40.56 |  |
| *MNPs + IMH* | | | | |
| 48.55 | 1.46·10^-4^ | 8.48 | 40.77 |  |

**Supplementary Table S3.** CT and SWE properties of a designed breast phantom, Median (Q1–Q3)

| **Region of interest** | **Radiodensity, HU** | **Apparent stiffness, kPa** |
| --- | --- | --- |
| Breast-mimicking region | 79.00  (65.00–93.00) | 26.09  (12.59–35.56) |
| Tumour-mimicking region | 49.00*  (32.25–58.50) | 8.18*  (6.11–9.50) |
| Tumour-mimicking region + MNPs | 3115.00*^#^  (2498.25–3932.00) | 187.59*^#^  (113.99–292.26) |

^*^statistically significant difference from breast tissue-mimicking region, p < 0.05;

^#^statistically significant difference from tumour-mimicking region, p < 0.05.
